# Human CD4+CD25+ Regulatory T Cells Are Sensitive to Low Dose Cyclophosphamide: Implications for the Immune Response

**DOI:** 10.1371/journal.pone.0083384

**Published:** 2013-12-23

**Authors:** Daniel Heylmann, Martina Bauer, Huong Becker, Stefaan van Gool, Nicole Bacher, Kerstin Steinbrink, Bernd Kaina

**Affiliations:** 1 Department of Toxicology, University Medical Center, Mainz, Germany; 2 Pediatric Neuro-Oncology, University Hospitals, Leuven, Belgium; 3 Department of Dermatology, University Medical Center, Mainz, Germany; Istituto Superiore di Sanità, Italy

## Abstract

Regulatory T cells (Treg) play a pivotal role in the immune system since they inhibit the T cell response. It is well known that cyclophosphamide applied at low dose is able to stimulate the immune response while high dose cyclophosphamide exerts inhibitory activity. Data obtained in mice indicate that cyclophosphamide provokes a reduction in the number of Treg and impairs their suppressive activity, resulting in immune stimulation. Here, we addressed the question of the sensitivity of human Treg to cyclophosphamide, comparing Treg with cytotoxic T cells (CTL) and T helper cells (Th). We show that Treg are more sensitive than CTL and Th to mafosfamide, which is an active derivative of cyclophosphamide, which does not need metabolic activation. The high sensitivity of Treg was due to the induction of apoptosis. Treg compared to CTL and Th were not more sensitive to the alkylating drugs temozolomide and nimustine and also not to mitomycin C, indicating a specific Treg response to mafosfamide. The high sensitivity of Treg to mafosfamide resulted not only in enhanced cell death, but also in impaired Treg function as demonstrated by a decline in the suppressor activity of Treg in a co-culture model with Th and Helios positive Treg. Treatment of Treg with mafosfamide gave rise to a high level of DNA crosslinks, which were not repaired to the same extent as observed in Th and CTL. Also, Treg showed a low level of γH2AX foci up to 6 h and a high level 24 h after treatment, indicating alterations in the DNA damage response. Overall, this is the first demonstration that human Treg are, in comparison with Th and CTL, hypersensitive to cyclophosphamide, which is presumably due to a DNA repair defect.

## Introduction

CD4+CD25+ regulatory T cells (Treg) play a key role in suppressing immune responses. Treg prevent inflammation and autoimmune disorders by inhibiting the activity of T effector cells including CD4+ T helper cells (Th) and CD8+ cytotoxic T cells (CTL) [1]. Diverse mechanisms are involved in the suppression of the immune system by Treg. Thus, Treg produce cytokines such as TGF-β, IL-10 and IL-35 that inhibit effector T cells. They can also kill effector T cells by cytolysis and perforin mediated killing triggered by granzyme secretion. Additionally, Treg also target dendritic cells (DCs) by recognizing MHC class II molecules via the CD4 homologue LAG3 (lymphocyte activating gene 3), thereby suppressing DC maturation and their ability to stimulate the immune system. Treg also express CTLA4 (cytotoxic T-lymphocyte antigen 4), which interacts with CD80/CD86 on the surface of DC. This leads to the induction of indolamine 2,3-dioxygenase, which leads to the production of immuno-modulating pro-apoptotic factors resulting from tryptophan degradation. Furthermore, a tryptophan-deprived environment provokes killing of effector T cells [2], [3]. If these mechanisms are out of balance, negative effects such as autoimmunity and uncontrolled immune responses to pathogens or allergens will be resulting. At the other side, it may cause tolerance to cancer cells. Treg inhibit the antitumoral immune activity, thereby promoting tumor progression [1], [4]. It is important to note that tumor tissue is often infiltrated with Treg, which is supposed to attenuate the host immune response directed against the tumor tissue [5].

**Figure 1:**
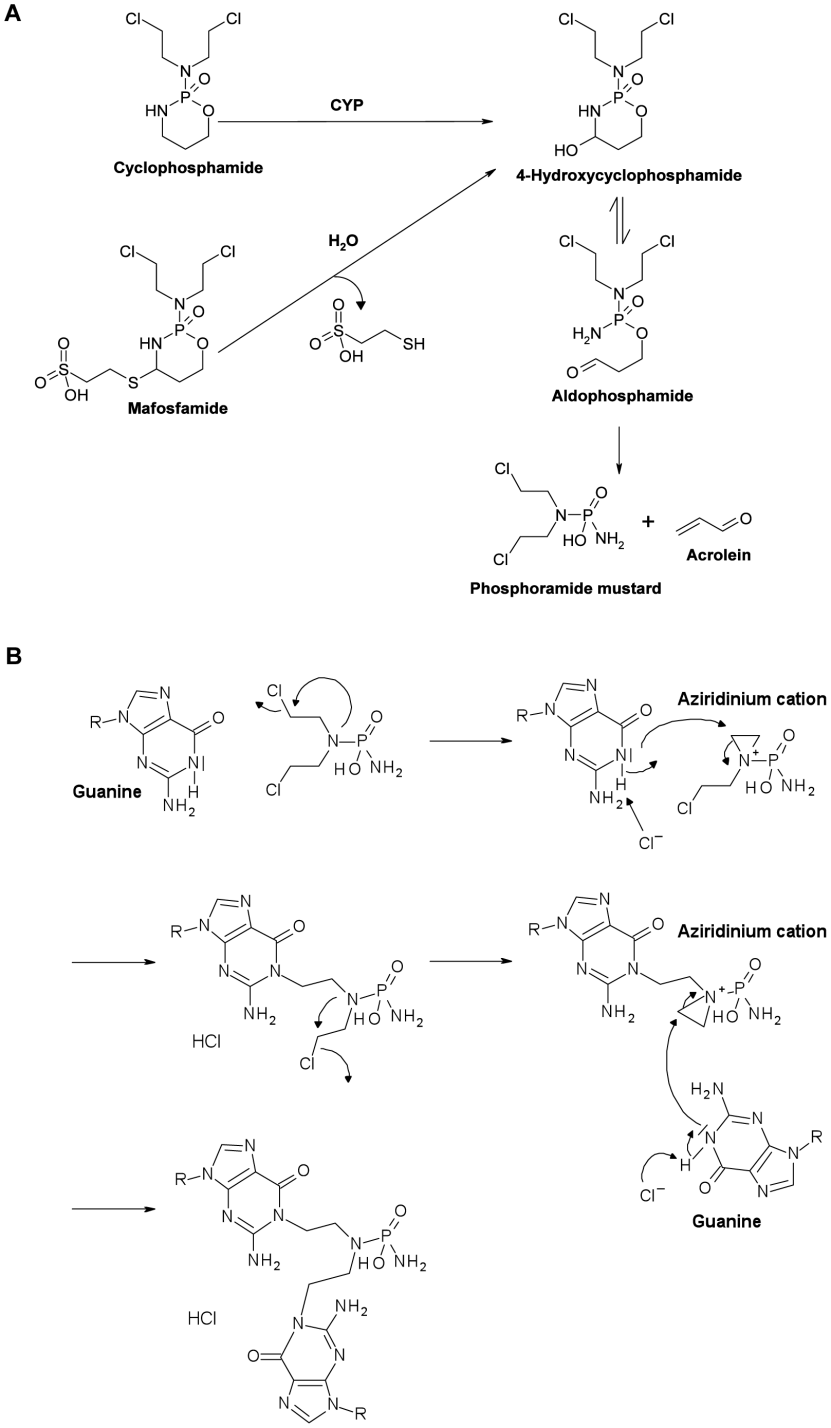
Mode of action of cyclophosphamide and mafosfamide. A, Decay into the active form, phosphoramide mustard. B, Binding to guanine and formation of guanine-guanine ICL. Structural formulas are drawn with Accelrys Draw 4.1 SP1 (Accelrys, Inc., San Diego, CA).

Cyclophosphamide is being used to treat various types of cancer and autoimmune diseases. High dose cyclophosphamide leads to immunosuppression, whereas low dose cyclophosphamide was shown to enhance the immune response [6], [7]. The underlying reason is still a matter of speculation. However, it is important to understand these opposite effects of low and high dose cyclophosphamide because immunostimulation is desired in cancer therapy, but not in the treatment of autoimmune diseases and, the other way around, immunosuppression is desired in treatment of autoimmune diseases, but not in cancer therapy. Cyclophosphamide is metabolized by the cytochrome P450 system that generates 4-hydroxycyclophosphamide. 4-hydroxycyclophosphamide is unstable and becomes decomposed into the active compound phosphoramide mustard, which alkylates DNA at the N7 position of guanine forming DNA interstrand crosslinks (ICL) that are supposed to be the ultimate cytotoxicity triggering lesions (Fig. 1A,B) [6], [8]. Mafosfamide is a derivative of cyclophosphamide, which is active without metabolization and, therefore, suitable for *in vitro* studies [9]. The bioavailibility of cyclophosphamide and 4-hydroxycyclophosphamide was investigated in pharmacokinetic trials with cancer patients and patients with inflammatory diseases receiving i.v. 0.7 g/m^2^ cyclophosphamide. The plasma concentration of cyclophosphamide ranged between 12 and 18 µg/ml approaching a level of <2.5 µg/ml after 24 h [8], [10]. The 4-hydroxyclophosphamide levels ranged from 0.4 to 0.1 µg/ml 24 h after administration [8], [10]. In the *in vitro* experiments described here the dose of mafosfamide was in the range of 0.5–2 µg/ml, which corresponds to a low serum level of cyclophosphamide.

Experiments with mice demonstrated that low dose cyclophosphamide selectively depletes circulating Treg and, at the same time, enhances the immune response [11], [12]. Decreasing numbers of Treg by exposure of mice with low dose cyclophosphamide resulted in an imbalance between antitumor natural killer T cells and DCs. This was accompanied by an attenuated progression of multiple myeloma in mice [7], [13]. Treg depletion by cyclophosphamide also improved the outcome of tumor vaccination against colon carcinomas in rats [14]. Furthermore, a phase II study of renal cancer patients demonstrated that a single dose cyclophosphamide (300 mg/m^2^) decreased the number of Treg and extended the survival of patients after immunotherapy [15]. In another study with cancer patients receiving 50 mg cyclophosphamide orally twice a day every two weeks, a selective depletion of Treg was observed after 30 days of treatment in peripheral blood concomitant with an increased effector T cell response. Higher doses of cyclophosphamide (200 mg/day) revealed no specificity in Treg killing and impacted all lymphocyte subpopulations [16]. Metronomic cyclophosphamide treatment also caused short-term Treg reduction in the peripheral blood of breast cancer patients along with an enhanced expansion of CD4+ and CD8+ T effector cells [17]. A low level of ATP and glutathione was proposed to explain the specific Treg response towards cyclophosphamide as low glutathione reduces the detoxification capacity of cells [18].

The finding of a change in the number of Treg in the peripheral blood following low dose cyclophosphamide, which was observed in animal settings and patients, leads to the question of whether Treg are hypersensitive to genotoxic agents, notably alkylating drugs such as cyclophosphamide that induce ICL. To our knowledge, the sensitivity of Treg to genotoxic agents has not yet been studied *in vitro*. Here, we elucidated the killing response of Treg and compared them with CTL and Th cells following treatment with the active cyclophosphamide analogue, mafosfamide. We also studied functional aspects such as Treg suppressor activity and Helios expression. The data revealed that Treg are indeed hypersensitive to mafosfamide, which pertains to the killing effects and impaired suppressor activity. It seems to be a specific response of Treg to this type of alkylating agent, which is likely related to impaired DNA repair.

## Materials and Methods

### Culture Medium and reagents

X-VIVO-15 (BioWhittaker, Walkersville, MD) supplemented with 10% autologous plasma was used for the culture of T cells (Treg, Th and CTL). Cells were incubated at 37°C and 5% CO_2_. Mafosfamide was a gift from ASTA Medica (Frankfurt, Germany), nimustine was from Sigma-Aldrich (St- Louis, Germany), temozolomide from Schering-Plough, mitomycin C (MMC) and acrolein from Sigma-Aldrich. The drugs were solubilized in sterile distilled water except temozolomide, which was dissolved in DMSO and further diluted in distilled water.

### Isolation of cells

CD4+CD25+ (Treg), CD4+ (Th) and CD8+ (CTL) cells were isolated from peripheral blood mononuclear cells (PBMC) using the Regulatory T Cell Isolation Kit and anti-CD4 and anti-CD8 beads from Miltenyi Biotec (Bergisch Gladbach, Germany) according to the manufacturer's protocol. PBMC were isolated by Ficoll–Hypaque density gradient centrifugation from buffy coats of blood donors obtained from the blood bank of the University Medical Center Mainz. All cell preparations were checked as to cell surface markers by flow cytometry using anti-CD25-PE, anti-CD4-FITC and anti-CD8-APC (Miltenyi Biotec, Bergisch Gladbach, Germany), and preparations that were of low purity (<75%) were excluded. The Treg population displayed the Foxp3 signal, as determined by flow cytometry.

### Quantification of apoptosis

Apoptosis was measured by flow cytometry. *Sub-G1 assay*: After treatment with the agents CD4+25+, CD4+ and CD8+ cells were washed in PBS and fixed in 70% ethanol for a minimum of 30 min at −20°C. DNA in the cells was stained with propidium iodide (PI) (16.5 µg/ml) in PBS after RNase (0.03 µg/ml) digestion. For each sample, 10^4^ cells were analyzed on a FACSCalibur device (Becton Dickinson, Heidelberg, Germany). The number of apoptotic cells per sample was calculated using the computer program WinMDI 2.9 (Joseph Trotter, Salk Institute for Biological Studies, La Jolla, CA.).

Annexin V/PI assay: The annexin V/PI assay distinguishes between early apoptotic cells and late apoptotic/necrotic cells by using annexin V/PI double staining of unfixed cells. Cells were suspended in 50 µL binding buffer (10 mM Hepes, pH 7.4, 140 mM NaCl, 2.5 mM CaCl, 0.1% BSA) and annexin V-FITC (2.5 µl; Miltenyi Biotec, Bergisch Gladbach, Germany) was added to each sample. After 15 min incubation in the dark, 430 µL binding buffer and 1 µg/ml PI per sample were added. The flow cytometric analysis was carried out by using a FACSCalibur device (Becton Dickinson, Heidelberg, Germany). For each sample, 10^4^ cells were analyzed. Calculation of apoptotic/necrotic cell populations was performed using the computer program WinMDI 2.9 (Joseph Trotter, Salk Institute for Biological Studies, La Jolla, CA) or BD FACSDiva software (Becton Dickinson).

### *In vitro* suppressor assay

Suppressor assays were performed in 96-well round-bottom plates (Greiner Bio-One, Frickenhausen, Germany) in a final volume of 200 µl/well of X-VIVO-15 (BioWhittaker, Walkersville, MD). Treg were pretreated with or without mafosfamide for 24 h. The CD4^+^ (Th) and CD4+CD25+ (Treg) cells were plated at 1×10^5^/well alone or in combination in triplicate. The cells were cocultured at the ratios 1∶0 and 1∶1 (CD4+∶CD4+CD25+). Stimulation was carried out with purified NA/LE mouse anti-human CD3 monoclonal antibody (BD Pharmingen, Heidelberg, Germany; 0,1 µg/well) and allogenic irradiated (50 Gy) PBMCs (3×10^5^/well). On day 3, 0.5 µCi of ^3^H-thymidine (Perkin Elmer, Waltham, MA) was added for 16 h of culture. The cells were then harvested and assessed for uptake of the labeled thymidine by liquid scintillation. Suppressive capacity of Treg in percent was calculated by using the formula: [1- (mean cpm (mixed cell culture; 1∶1)/mean cpm (effector cells; 1∶0)]×100. *p<0.05 for comparison of suppressive capacity of treated Treg and untreated Treg determined by the t-test. Data of at least three independent experiments are pooled.

### Intracellular staining for flow cytometry

For intracellular staining of the transcription factor Helios, 250 000 Treg, Th or CTL were seeded after magnetic bead isolation per 0.5 ml medium and treated with Mafosfamide. After 48 h, the cells were harvested and pelletized. Dead cells were directly stained with fixable viability dye eFluor®450 (eBioscience, San Diego, CA) using protocol c: *staining dead cells with ebioscience*® *fixable viability dyes* (eBioscience, San Diego, CA). Then protocol b: *one step protocol for intracellular (nuclear) proteins* (eBioscience, San Diego, CA) was used along with Foxp3 staining buffer set (eBioscience, San Diego, CA). The cells were stained with 0.125 g anti-Helios-FITC (eBioscience, San Diego, CA) per sample in 100 µl 1× permeabilization buffer (eBioscience, San Diego, CA) for 30 min. Analysis was performed by flow cytometry (BD, Heidelberg, Germany, FACS CantoII) and BD FACSDiva software. p*<0.05 for comparison of treated and untreated T cells determined by the Post-Hoc Bonferroni test (SPSS statistics, IBM, Armonk, NY). Data of three independent experiments are pooled.

### Comet ICL assay

The detection of interstrand crosslinking was investigated using a modification of single cell gel electrophoresis (comet assay) as described previously [19]. Cells were treated with mafosfamide and harvested after 4 h and 24 h. All mafosfamide-treated samples and one control were subjected to 8 Gy γ-irradiation to induce random strand breakage, one unirradiated control was also included. The cells were analysed by alkaline single cell gel electrophoresis (comet assay). The presence of ICLs retards migration of the irradiated DNA during electrophoresis, resulting in reduced tail moment compared to control cells. The amount of ICLs was therefore determined by comparing the tail moment of the irradiated mafosfamide-treated samples with irradiated untreated samples and unirradiated untreated controls. The level of interstrand crosslinks (ICL) [20] is proportional to the decrease in tail moment (DTM) in the irradiated drug treated sample compared to the irradiated untreated control. The % ICL was calculated using the following formula % DTM = [1−(TMDIR−TMCU)/(TMCIR−TCU)]×100, where TMDIRs the mean tail moment of the mafosfamide treated irradiated sample, TMCIR is the mean tail moment of the irradiated control sample and TMCU is the mean tail moment of the unirradiated control sample. The unhooking of DNA interstrand cross-links was expressed as percent unhooking, which was calculated using the formula % unhocking of DNA ICLs at 24 h = [(% DTM at 4 h -% DTM at 24 h)/% DTM at 4 h]×100. *p<0.05 and **p<0.01 for comparison of Treg, Th and CTL determined by the t-test. Data of at least three independent experiments are pooled.

### Immunostaining of cells

Prior to immunostaining, cells were fixed with methanol, acetone and paraformaldehyde, then blocked with normal goat serum (Invitrogen, Carlsbad, CA). The phosphorylation of H2AX was detected with monoclonal (81299)/polyclonal (11174) rabbit anti-phospho-γH2AX (Ser139) (Abcam, Milton, GB) and then with the second antibody goat anti-rabbit F(ab')_2_ conjugated with AlexaFluor488 (Invitrogen, Carlsbad, CA). Nuclei were dyed with ToPro3 (Invitrogen, Carlsbad, CA) for 15 min in the dark and the samples stocked with VectaShield mounting medium for fluorescence (Vector laboratories, Burlingame, CA) and sealed with nail polish. Analysis was performed at LSM710 (Carl Zeiss, Oberkochen, Germany). Mean intensity of γH2AX foci was measured using the ZEN software (Carl Zeiss, Oberkochen, Germany). Mean intensities of γH2AX foci per cell of at least three different experiments are pooled.

## Results and Discussion

**Figure 2:**
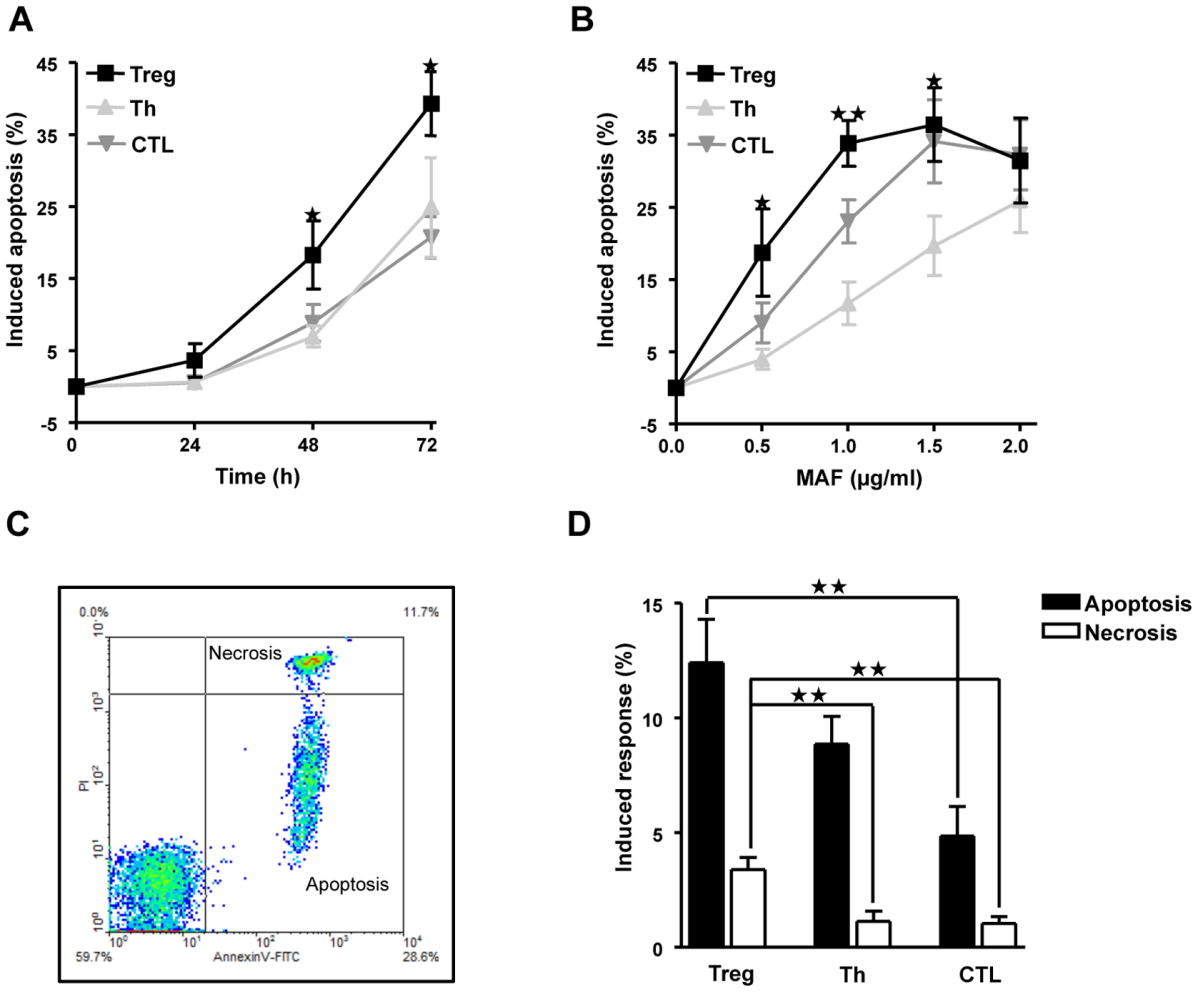
Cyctotoxicity of mafosfamide in Treg, Th and CTL. A, Time course of the induction of apoptosis, which was determined by subG1 quantification. Cells were treated with a dose of 0.5 µg/ml mafosfamide and cells were subjected to flow cytometry at the times indicated. B, Dose response of apoptosis induction in Treg, Th and CTL. The fraction of subG1 was determined 48 h after mafosfamide treatment. The asteriks label the measure points that show a significant difference to Th and CTL, except for the 1.5 µg/ml MAF level, for which significance was obtained only against Th. C, Amount of apoptosis (annexinV positive, PI negative fraction) and necrosis (annexinV positive, PI positive fraction), as determined by the annexinV/PI flow cytometry assay. Treg were treated with 0.5 µg/ml mafosfamide. D, Yield of apoptosis and necrosis in Treg, Th and CTL treated with 0.5 µg/ml. Cells were harvested 24 h after the onset of treatment. Data are the mean of at least three independent experiments.

To compare the sensitivity to mafosfamide of Treg with Th and CTL, we isolated T cell populations for each experiment freshly from buffy coats of healthy volunteers. After a day of cultivation, mafosfamide was added to the medium and cells were incubated for the indicated time periods. As shown in Fig. 2A, cells start to die by apoptosis one day after treatment and continue to undergo apoptosis 48 and 72 h following treatment. The frequency of apoptosis linearly increased with dose up to 1 µg/ml mafosfamide (48 h fixation), with Treg showing clearly the strongest response. With a dose of 1,5 µg/ml saturation was reached and apoptosis did not further increase in Treg. With a high dose of 2 µg/ml the other T cell types approached the killing level of Treg (Fig. 2B). Thus the hypersensitivity of Treg is most obvious at low mafosfamide concentrations (≤1,5 µg/ml). Mafosfamide induced mainly apoptosis while necrosis was only marginally observed in Treg, as determined by annexin V/PI double staining and flow cytometry (Fig. 2C for a representative plot and Fig. 2D for quantification). The same was true for Th and CTL, albeit at a lower level. The difference in the amount of apoptosis and necrosis induced by mafosfamide (0.5 µg/ml) in Treg compared to Th and CTL was significant (Fig. 2D).

**Figure 3:**
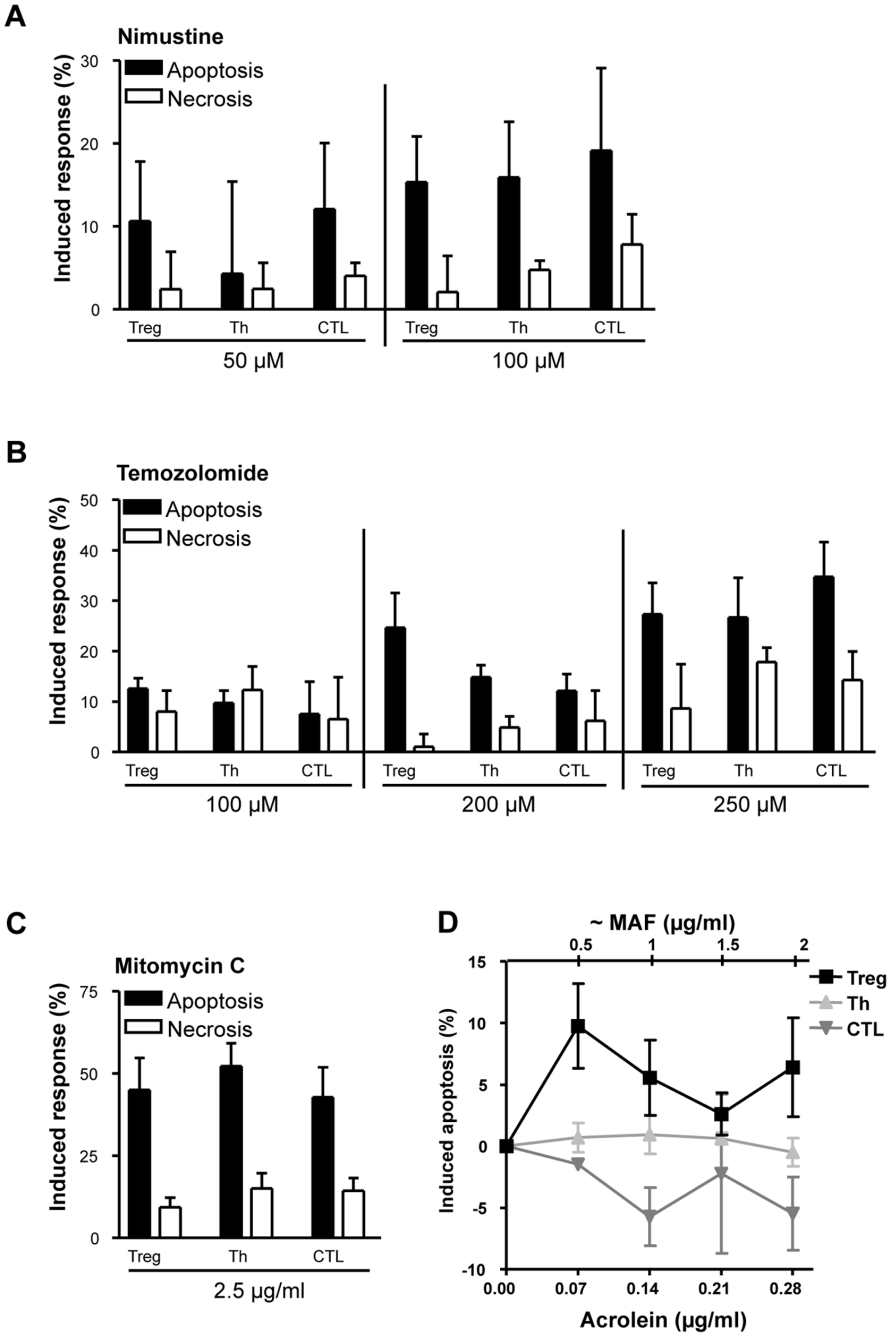
Level of apoptosis and necrosis (determined by the annexinV/PI assay) in Treg, Th and CTL following treatment with different agents. A, apoptosis and necrosis following treatment with 50 µM (13,6 µg/ml) and 100 µM (27,3 µg/ml) nimustine (ACNU). Fixation occurred 72 h after treatment. B, apoptosis and necrosis following treatment with 100 µM (19,4 µg/ml), 200 µM (38,8 µg/ml) and 250 µM (48,5 µg/ml) temozolomide. Fixation occurred 72 h after treatment. C, apoptosis and necrosis following treatment with mitomycin C. Fixation occurred after 48 h. D, apoptosis (determined by subG1) induced by acrolein in Treg, Th and CTL after 48 h. The abscissa points to the dose of acrolein and, on the top, the equivalent dose of mafosfamide, which is in the range of 0–2 µg/ml (compare with Fig. 2B). Data are the mean of at least three independent experiments. The solvent (DMSO for temozolomide) and the aqueous controls were not toxic if given without the drug.

Cyclophosphamide resp. mafosfamide is an alkylating agent. Therefore, we wondered whether Treg are hypersensitive to other alkylating drugs as well. To this end we treated the cells with the chloroethylating agent nimustine (ACNU) and the methylating drug temozolomide and determined by annexin/PI the yield of apoptosis and necrosis. We observed that Treg are not more sensitive to these agents compared to Th and CTL (Fig. 3A for nimustine and 3B for temozolomide). We also determined the sensitivity to mitomycin C (MMC), which is a crosslinking agent [21], and also observed no significant difference in the response (Fig. 3C and data not shown). It therefore appears that the hypersensitivity of human Treg is a specific response to mafosfamide resp. cyclophosphamide.

Since degradation of mafosfamide yields the carbamoylating metabolite acrolein (Fig. 1A), which damages proteins [22] and thus could contribute to the killing effects observed, the toxicity of acrolein was determined. Treg, Th and CTL were treated with increasing doses of acrolein, in stoichiometric equal amounts to mafosfamide, and the subG1 fraction was determined 48 h later. The data shows that acrolein induces only slight apoptosis (<10%) and there was no dose-dependent increase in the yield of apoptosis, neither in Treg nor Th and CTL in a dose range that approximates the killing dose level of mafosfamide (0.5–2 µg/ml) (Fig. 3D). As cell death induced by acrolein was low and did not exceed the level induced by mafosfamide, we infer that acrolein does not play a major role in the killing response in this experimental system.

**Figure 4:**
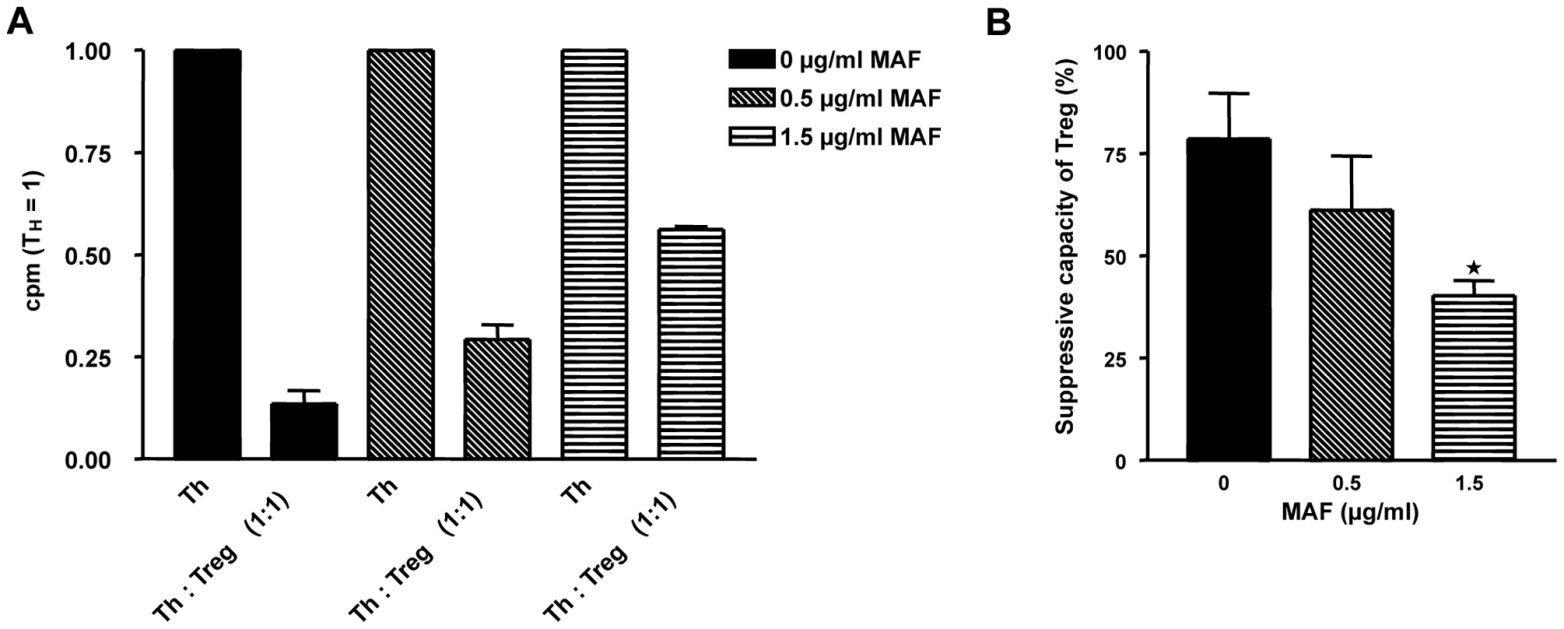
Effect of mafosfamide on the Th suppression activity of Treg. A, Th were co-incubated with the same amount of mafosfamide-pretreated Treg (1∶1), stimulated with antiCD3 antibody and allogenic high-dose irradiated PBMC, and thymidine incorporation was measured in the cell fraction. Treg were not treated or pre-treated with 0.5 and 1.5 µg/ml mafosfamide 24 h before co-cultivation with Th. Co-cultivation and stimulation occurred for 72 h, and thymidine was added to the medium 16 h before cell harvest. The thymidine incorporation in Treg was taken into account by subtracting the thymidine incorporation (cpm/cell) in mono-cultivated and stimulated Treg from the total population. Data were corrected for cytotoxicity i.e. the dead cell Treg fraction was not taken into account. Data are the mean of three independent experiments. B, Relative suppression activity of Treg. Data are from Fig. 4B. * p<0.05. We should note that in this assay the data were corrected as to cytotoxicity.

As Treg are sensitive to mafosfamide, it is conceivable that mafosfamide impairs their function, i.e. their Th suppressive activity. This was checked in a suppressor assay analyzing the effect on activated Th cells. As shown in Fig. 4A, co-cultivation of Th with Treg strongly reduced DNA replication of Th (determined by the thymidine incorporation assay). Co-cultivation of mafosfamide pretreated Treg with Th reduced dose-dependently the suppression of Th, the quantification is shown in Fig. 4B. The data suggests that non-repaired mafosfamide-induced damage impairs the function of Treg.

**Figure 5:**
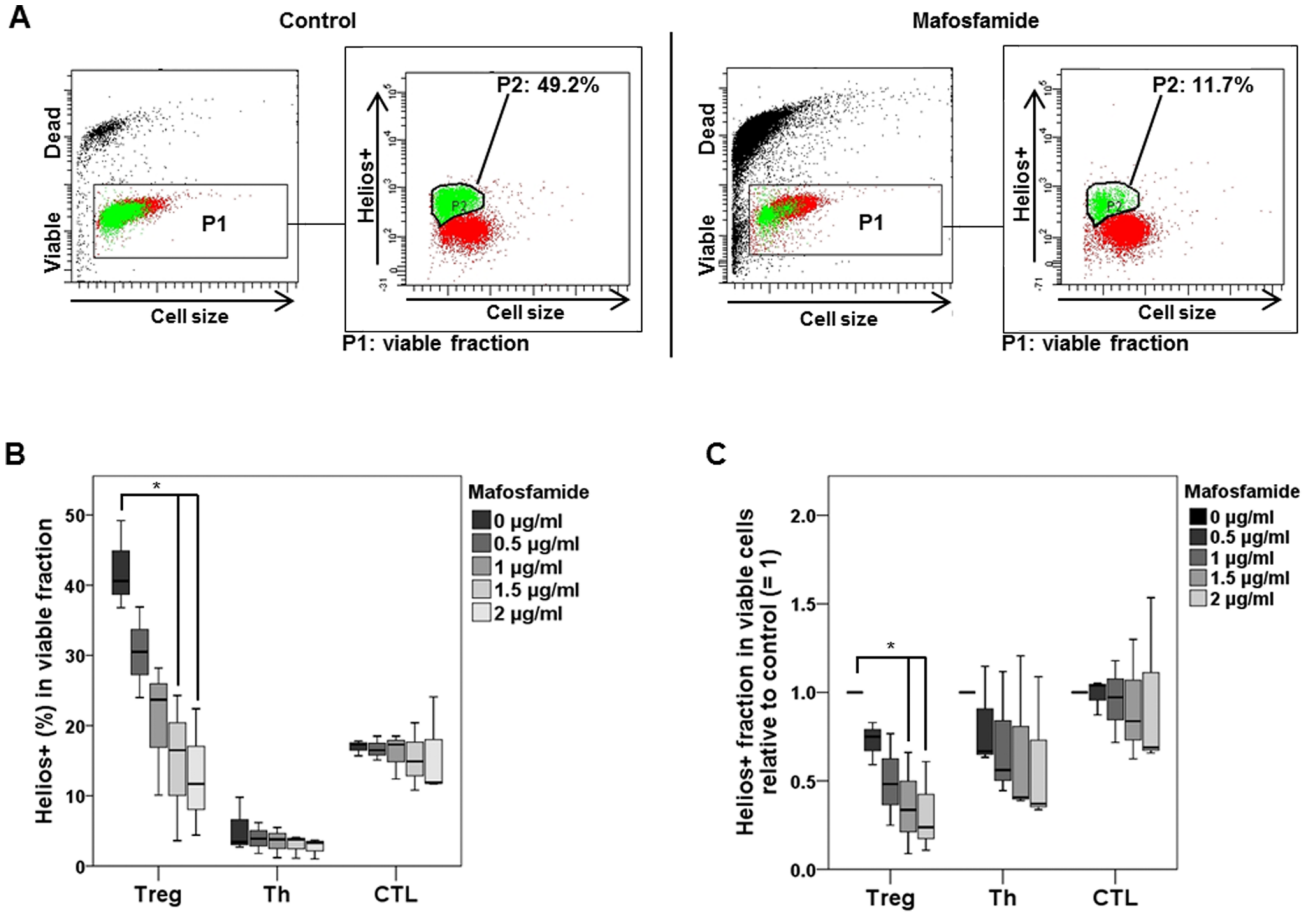
Helios expression in viable Treg. A, Dot plots of control and mafosfamide (2 µg/ml, 48 h) treated Treg. The living fraction was gated (P1) and Helios+ cells were marked green in this fraction (designed as P2). Left panel shows representative graphs of untreated Treg; right panel of treated Treg. B, Quantification of Helios expressing Treg, Th and CTL determined 48 h after treatment with 2 µg/ml mafosfamide. C, Quantification of data with the non-treated control set to 1. * p<0.05 significance.

To consolidate the data, we studied the effect of mafosfamide on Helios expression in Treg, Th and CTL. Helios, a DNA binding protein belonging to the *Ikaros* group of transcription factors, is expressed specifically in T cells, notably Treg and CTL. Its expression is considered as a marker of T cell proliferation and T cell activity, notably in Treg [23]. Therefore, we wondered whether mafosfamide treatment impacts Helios expression. As shown in Fig. 5A, in the non-treated control 49.2% of viable Treg were positive for the Helios signal (Helios+) whereas in mafosfamide treated Treg only 11.7% were Helios+. The data were quantified and shown in Fig. 5B for increasing mafosfamide concentrations 48 h after treatment. Treg show an about two times higher Helios+ living fraction than CTL, and Th show the lowest basal level of Helios expression. Thus, the basal levels (untreated cells) of Helios+ are similar to a previous report [23]. After treatment with mafosfamide, a clear decrease in the fraction of Helios+ Treg cells was observed, which was most significant after 1.5 and 2 µg/ml mafosfamide. A decline in the Helios+ fraction following mafosfamide was not observed for Th and CTL cells. In Fig. 5C data are shown with the control set to 1, indicating a slight but insignificant decrease in the level of Helios+ cells for Th and CTL, but a significant decline for Treg. The data support the hypothesis that mafosfamide exerts influence on the function of Treg by decreasing the expression of Helios (the number of Helios+ Treg declined), which overall contributes to the suppression of Treg function.

**Figure 6:**
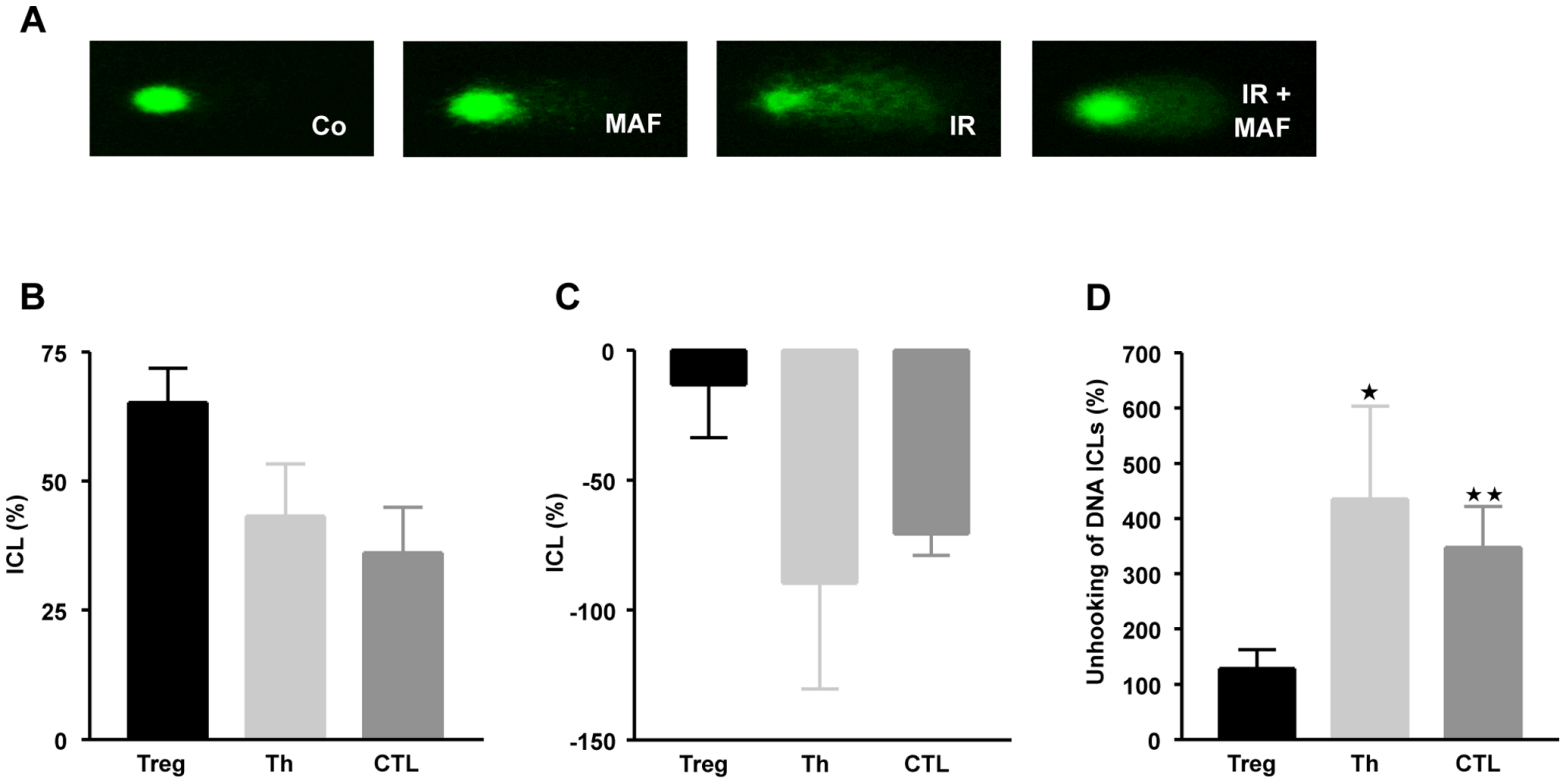
Crosslink induction and repair in Treg, Th and CTL. A, Representative comets of cells not treated (control), mafosfamide (20 µg/ml) treated, gamma-ray treated (IR, 8 Gy) and IR plus mafosfamide treated. B, Quantification. ICL (%) was defined as decrease in tail moment as described in material and methods, 4 h after treatment. C, ICL (%) 24 h after treatment. D, Relative repair of ICL, which is given by the relative ICL level 24 h after treatment in relation to the level 4 h after treatment. Data are the mean of at least three independent experiments. * p<0.05; **p<0.01 significance.

Cyclophosphamide and its active derivative mafosfamide induce DNA interstrand crosslinks (ICL), which are thought to be majorly responsible for the cytotoxic response of the cells [9]. Therefore, we posit that Treg are impaired in the repair of cyclophosphamide induced ICL. To assess whether Treg are impaired in the repair of ICL, we took advantage of the modified alkaline single cell gel electrophoresis assay (comet assay) and observed that the amount of induced ICL in Treg was higher than in Th and CTL (Fig. 6A for representative stainings, and Fig. 6B for the relative ICL level 4 h after addition of mafosfamide to the medium). Interestingly, 24 h after mafosfamide treatment the ICL level was reduced in Th and CTL, but not in Treg (Fig. 6C). The difference between induced and after a recovery period observed ICL provides a measure of ICL repair, which is given in Fig. 6D. It shows that a significant fraction of ICL is repaired in Th and CTL, while in Treg the amount of ICL remained nearly unchanged. The data indicate that Treg are impaired in the repair of mafosfamide induced ICL.

**Figure 7:**
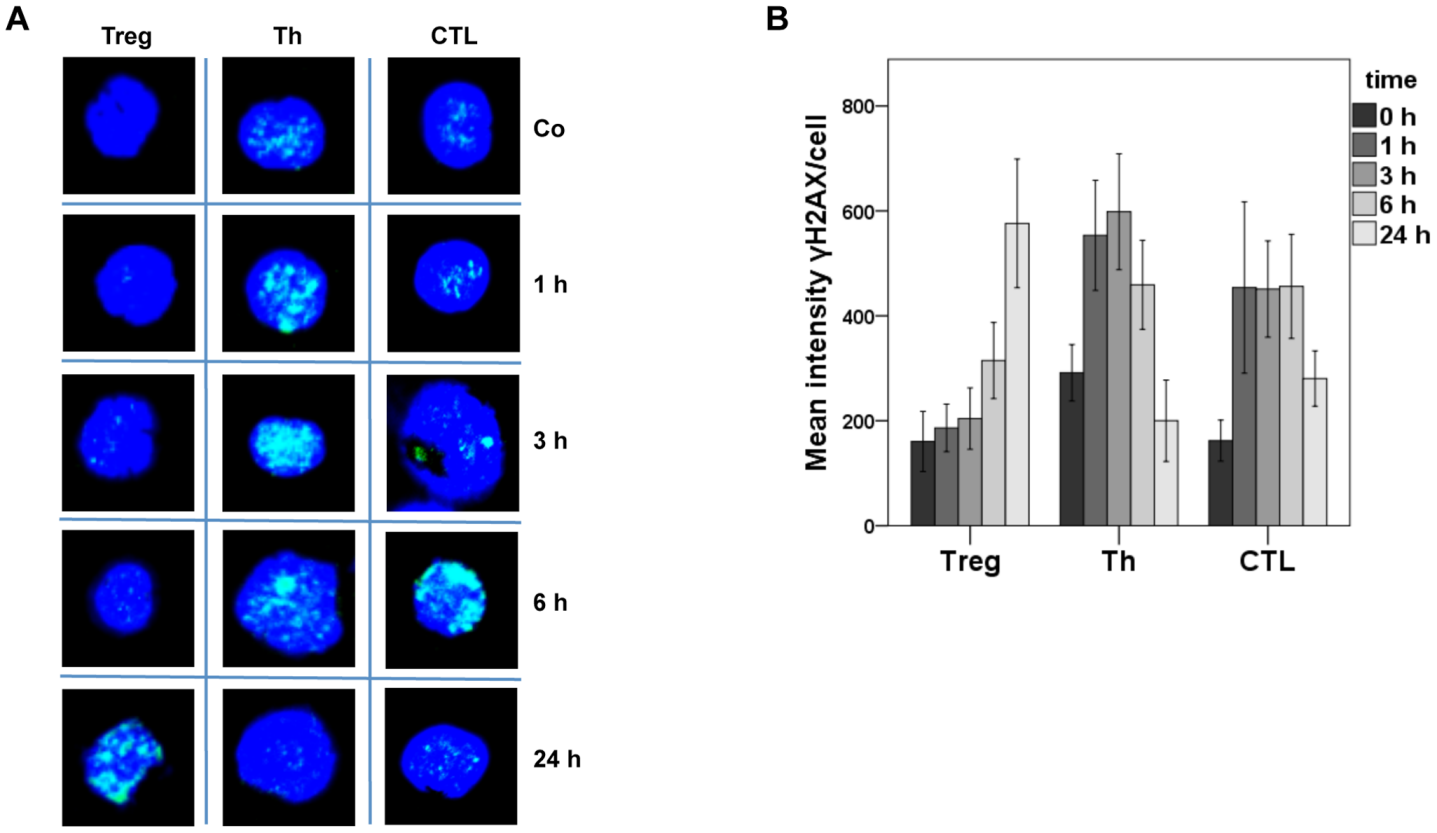
γH2AX staining of Treg, Th and CTL not treated (control) and treated with mafosfamide. A, Representative stainings 1 up to 24(1 µg/ml). Blue, nuclear staining with ToPro 3. Green, staining of γH2AX. B, Quantification for Treg, Th and CTL at different times after treatment. Mean intensities of γH2AX foci per cell of at least three independent experiments are pooled (standard error).

Non-repaired DNA lesions may lead to DNA double-strand breaks (DSB), which are most critical cytotoxic lesions [24], [25]. DSB trigger the formation of phosphorylated histone 2AX (γH2AX), which can therefore be used as a marker for the presence of DSB. In Fig. 7A, representative stainings for γH2AX are shown, demonstrating that 1, 3 and 6 h after treatment there was less γH2AX staining in Treg than in CTL and Th. On the other hand, 24 h after mafosfamide treatment Treg responded with a much higher amount of γH2AX than CTL and Th (for quantification see Fig. 7B). The low level of γH2AX staining at early times after the onset of mafosfamide treatment can be explained by a lack of DNA damage incision activity in Treg, which induces repair intermediates that trigger γH2AX phosphorylation. Since repair occurred 24 h after treatment in CTL and Th, γH2AX are disappearing while in Treg the DNA damage response became activated due to non-repaired ICL. Thus, the high amount of γH2AX in Treg 24 h after treatment is compatible with the idea that Treg are impaired in the repair of mafosfamide-induced ICL.

Reduction of the amount of Treg (which was in most cases compared in relation to Th) in the peripheral blood of cyclophosphamide-treated individuals has been observed both in human and mice [11], [12], [16], [17]. Here we demonstrate for the first time that isolated human Treg are hypersensitive to cyclophosphamide (using the active form of the drug, mafosfamide) as compared to Th and CTL. We also show that the agent impairs the function of Treg, as demonstrated by the Treg suppressor assay (suppression of Th proliferating activity) and decline of Helios expression in viable Treg. Suppression of Treg proliferation activity was recently observed in renal cancer patients treated with a single low dose of cyclophosphamide [15]. Our data obtained on purified Treg strongly support this observation.

It is reasonable to hypothesize that the enhanced immune response observed after low dose cyclophosphamide in patients is due to the high killing response of Treg caused by impaired repair of cyclophosphamide-induced DNA damage. In a previous report we have shown that human monocytes are more sensitive than dendritic cells and macrophages to monofunctional alkylating agents, which was shown to be the result of defects in base excision and DSB repair [26], [27]. The findings presented here may be taken to indicate that another blood cell population displays a DNA repair defect as well, which pertains specifically to a pathway involved in ICL repair. The defect at molecular level still needs to be identified.

Treg have become an important player in regulating anticancer immune responses. Published studies describe a correlation between the number of blood or tumor-infiltrating Treg and poor prognosis [28]. Unfortunately, specific therapeutic approaches that target Treg are currently unavailable. However, cyclosphosphamide has emerged as a clinically feasible agent that can suppress the number and suppressive activity of Treg and may allow more effective antitumor immune responses. In clinical studies and murine tumor models, it was demonstrated that single and metronomic low dose administration of cyclophosphamide preferentially influences Treg function while other T cell populations remained unaffected [7], [12]. These observations are in line with the results of our study presented here. As the application of low dose cyclophosphamide did not induce strong cytotoxicity on all T cell subpopulations, but significantly reduced the number of Treg and its activity, low dose cylophosphamide may indeed be an innovative cancer therapeutic approach, aimed at activating the immune response. Further, we should note that cyclophosphamide needs metabolic activation in the liver by cytochrome P450, which can vary between individuals. Therefore, the administration of mafosfamide might be considered in this approach as well. Future studies should evaluate more carefully the effect of cyclophosphamide/mafosfamide on Treg in combination with other anticancer immunotherapy protocols (e.g. immune vaccination with dendritic cells) both in experimental settings and cancer patients.
